# Insights into the Novel Cardiac Biomarker in Acute Heart Failure: Mybp-C

**DOI:** 10.3390/life14040513

**Published:** 2024-04-16

**Authors:** Adriana Chetran, Minerva Codruţa Bădescu, Ionela Lăcrămioara Şerban, Ştefania Teodora Duca, Irina Afrăsânie, Maria-Ruxandra Cepoi, Bianca Ana Dmour, Iulian Theodor Matei, Mihai Ştefan Cristian Haba, Alexandru Dan Costache, Ovidiu Mitu, Corina Maria Cianga, Cristina Tuchiluş, Daniela Constantinescu, Irina Iuliana Costache-Enache

**Affiliations:** 1 Department of Internal Medicine I, Faculty of Medicine, University of Medicine and Pharmacy “Grigore T. Popa”, 700115 Iasi, Romania; adriana.ion@umfiasi.ro (A.C.); cepoi_maria-ruxandra@d.umfiasi.ro (M.-R.C.); bianca-ana-dmour@email.umfiasi.ro (B.A.D.); matei.theodor-iulian@d.umfiasi.ro (I.T.M.); mihai.haba@umfiasi.ro (M.Ş.C.H.); dan-alexandru.costache@umfiasi.ro (A.D.C.); ovidiu.mitu@umfiasi.ro (O.M.); irina.costache@umfiasi.ro (I.I.C.-E.); 2 Cardiology Clinic, “St. Spiridon” County Emergency Hospital, 700111 Iasi, Romania; 3 III Internal Medicine Clinic, “St. Spiridon” County Emergency Hospital, 700111 Iasi, Romania; 4 Department of Morpho-Functional Science II-Physiology, University of Medicine and Pharmacy “Grigore T. Popa”, 700115 Iasi, Romania; ionela.serban@umfiasi.ro; 5 Cardiovascular Rehabilitation Clinic, Clinical Rehabilitation Hospital, 700661 Iasi, Romania; 6 Department of Immunology, Faculty of Medicine, University of Medicine and Pharmacy “Grigore T. Popa”, 700115 Iasi, Romania; corina.cianga@umfiasi.ro (C.M.C.); daniela.constantinescu@umfiasi.ro (D.C.); 7 Immunology Laboratory, “St. Spiridon” County Emergency Hospital, 700111 Iasi, Romania; 8 Department of Microbiology, Faculty of Medicine, “Grigore T. Popa” University of Medicine and Pharmacy, 700115 Iasi, Romania; cristina.tuchilus@umfiasi.ro; 9 Microbiology Laboratory, “St. Spiridon” County Emergency Hospital, 700111 Iasi, Romania

**Keywords:** acute heart failure, biomarker, cardiac myosin-binding protein, MyBP-C, diagnosis, prognosis

## Abstract

(1) Background: Given its high cardiac specificity and its capacity to directly assess the cardiac function, cardiac myosin-binding protein (MyBP-C) is a promising biomarker in patients with acute heart failure (AHF). The aim of our study was to investigate the clinical utility of this novel marker for diagnosis and short-term prognosis in subjects with AHF. (2) Methods: We measured plasma levels of MyBP-C at admission in 49 subjects (27 patients admitted with AHF and 22 controls). (3) Results: The plasma concentration of MyBP-C was significantly higher in patients with AHF compared to controls (54.88 vs. 0.01 ng/L, *p* < 0.001). For 30-day prognosis, MyBP-C showed significantly greater AUC (0.972, *p* < 0.001) than NT-proBNP (0.849, *p* = 0.001) and hs-TnI (0.714, *p* = 0.047). In a multivariate logistic regression analysis, an elevated level of MyBP-C was the best independent predictor of 30-day mortality (OR = 1.08, *p* = 0.039) or combined death/recurrent 30-days rehospitalization (OR = 1.12, *p* = 0.014). (4) Conclusions: Our data show that circulating MyBP-C is a sensitive and cardiac-specific biomarker with potential utility for the accurate diagnosis and prognosis of AHF.

## 1. Introduction

Heart failure (HF) is a significant health concern worldwide, requiring prompt and accurate diagnosis for appropriate management [1,2]. Acute heart failure (AHF) is a potentially life-threatening condition and is one of the most common causes of emergency department presentation [3,4,5]. Despite great advances in diagnostic and treatment options, AHF is still associated with poor outcomes, high rates of readmission and mortality [3,5,6].

The diagnosis of AHF is a complex process that includes clinical evaluation, laboratory investigations (troponin, serum creatinine, electrolytes, blood urea nitrogen or urea, TSH, liver function tests, D-dimer, procalcitonin, arterial blood gas analysis, lactate), electrocardiogram (ECG), echocardiography, chest X-ray, lung ultrasound (LUS), pulse oximetry and natriuretic peptides (NPs) testing [3]. However, according to a study by Wong et al., the diagnosis of AHF is still missed in a range of 16% to 68%, due to symptoms similar to other conditions, especially respiratory diseases, or comorbidities such as atrial fibrillation (AF), chronic kidney disease (CKD), and chronic obstructive pulmonary disease (COPD) [7]. These data suggest that the current approach is imperfect and may benefit from improvement.

In recent years, the use of biomarkers has revolutionized the diagnosis and management of HF. They offer several advantages that support their use: the possibility of early detection, objective and quantifiable measurement, valuable prognostic information, personalized approaches to individual patient characteristics, allowing the longitudinal monitoring of disease progression and responses to treatment, cost-effectiveness and non-invasiveness [8]. Biomarkers, such as B-type natriuretic peptide (BNP), cardiac troponin (cTn), and soluble ST2, have emerged as valuable tools in HF diagnosis. These biomarkers aid in differentiating HF from other conditions, assessing disease severity, predicting prognosis, and guiding treatment decisions. The incorporation of these biomarkers into clinical practice has enhanced the diagnostic process, leading to improved patient outcomes and personalized management strategies for HF patients. Further research and advancements in biomarker utilization hold the potential for even greater precision and effectiveness in diagnosing and managing HF [9,10,11,12,13].

NPs, both B-type natriuretic peptide (BNP) and N-terminal pro-B-type natriuretic peptide (NT-proBNP), are currently the gold standard biomarkers recommended in the diagnosis of AHF as rule-out diagnostic tools [3,4]. NPs are secreted in response to increased cardiac wall stress, thus being a mark of increased intracardiac filling pressures, volume or pressure overload. They fulfill the role of counter-regulatory hormones causing vasodilation, reducing fibrosis and hypertrophy, and promoting natriuresis and diuresis [10,14]. Among the NPs, atrial natriuretic peptide (ANP) is released by the atrium, BNP is released by the ventricles and C-type natriuretic peptide (CNP) is released by the vascular endothelium. ANP has a very short half-life of 1 min with a very rapid clearance, making it unsuitable for diagnosing HF. By comparison, BNP has a half-life of 20 min, being eliminated from the circulation under the action of neprilysin. NT-proBNP is a BNP cleavage product, with a longer half-life of approximately 120 min, providing a more stable measurement [15]. Such studies as The Breathing Not Properly Study for BNP and ProBNP Investigation of Dyspnea in the Emergency Department (PRIDE) Study for NT-proBNP have confirmed the role of NPs in AHF diagnosis [16,17]. NPs have a negative predictive value according to current guidelines, thus recommending the use of these biomarkers only as an argument for excluding AHF in patients with acute dyspnea [3,4]. AHF diagnosis is unlikely if NPs have normal concentrations, and the cut-offs for AHF are: BNP < 100 pg/mL and NT-proBNP < 300 pg/mL. To improve diagnostic performance in HF, recent advances have identified an age-stratified model for NT-proBNP. The International Collaborative of NT-proBNP Study showed that the best predictive values for excluding HF diagnosis in an acute setting were NT-proBNP levels ≥450 pg/mL for ages less than 50, ≥900 pg/mL between 50 and 75 years, and ≥1800 pg/mL for ages greater than 75 years [18].

Apart from HF, several cardiac and non-cardiac conditions can cause elevated NPs concentrations, which support their interpretation only in relation to other clinical and paraclinical findings: acute coronary syndromes (ACS), pulmonary embolism (PE), myocarditis, valvular heart disease, cardiac contusion, atrial fibrillation, cardioversion, pulmonary hypertension, ischemic stroke, advanced age, renal dysfunction, liver dysfunction, COPD, anemia, severe infections, sepsis, and paraneoplastic syndrome [18].

Several limitations of NPs, including their low specificity and inability to directly assess the cardiac muscle injury independently of volume status, have prompted exploration for more biomarkers that could improve both diagnosis and prognosis.

Due to cardiac injury, detectable levels of cardiac troponin (cTn), especially high-sensitive troponin I (hs-TnI), have been observed in patients with AHF. Its role in this setting is to identify patients whose acute decompensated HF is due to acute myocardial infarction (AMI) or to predict patient outcomes [19,20,21]. Elevated end diastolic pressure, decreased perfusion of the endocardium, myocardial stretch, infiltrative disease states, and inflammation are the mechanisms involved in elevated troponin levels in AHF. The presence of detectable cTn concentrations appears to have important prognostic significance, being associated with increased risk of morbidity and mortality in both acute and chronic HF [19,20].

Soluble ST2 (suppression of tumorigenicity 2 protein) is a marker of ventricular remodeling and fibrosis, expressed by myocardial cells under stress [22]. In acute HF patients, ST2 levels were higher according to the PRIDE study, showing greater specificity than NPs, as the values were not influenced by age, renal insufficiency or obesity [23]. ST2 also showed a strong prognostic ability, with higher values significantly correlating with 1-year mortality risk [24]. Although currently the utility of ST2 is limited to research studies, it holds promise for future implementation in clinical practice as part of a multimarker approach.

In the search for additional biomarkers that may improve AHF management, studies were focused also on galectin-3, copeptin and GDF-15. Galectin-3 is a marker of inflammation and fibrosis that was associated with increased mortality and readmission in patients with AHF [25]. Copeptin is involved in fluid balance regulation and stress response, with promising results in AHF diagnosis. Higher levels of copeptin were present in patients with AHF compared with patients with non-cardiac dyspnea and were associated with increased mortality and adverse outcomes [26]. GDF-15 is secreted as a marker of inflammation, oxidative stress and cardiac remodeling, showing incremental prognostic value in patients with AHF, where it has been associated with increased mortality and adverse events [27]. Although the results of these biomarkers are promising, they are not currently included in routine clinical practice and need validation from further research.

Cardiac myosin-binding protein C (MyBP-C) is a large sarcomeric protein primarily found in the myocardium, that plays a critical role in cardiac contraction regulation by modulating the interaction between actin and myosin. During cardiac muscle contraction, myosin heads undergo a cyclic process of attachment to actin filaments, force generation, and detachment. MyBP-C acts as a molecular ruler, determining the optimal spacing between thick and thin filaments in the sarcomere by binding to myosin and actin. The protein extends from the C-zone of the sarcomere, where it binds to myosin, to the M-line region, where it interacts with titin and other sarcomeric proteins [28,29,30]. Its interactions with titin and troponin I and T contribute to the fine tuning of the contraction–relaxation cycle and enable the efficient transmission of the force generated by myosin to the sarcomere. The phosphorylation status of MyBP-C is a key determinant of its function. Phosphorylation by protein kinases modifies the protein’s interaction with myosin and actin, resulting in changes in cardiac contractility [31,32,33].

Pathogenic mutations in MyBP-C have been found to be the most common cause of hypertrophic cardiomyopathy (HCM) [33,34]. The understanding of the mechanisms underlying these mutations has provided valuable insights into the role of MyBP-C in cardiac physiology. Its abundance in cardiomyocytes and release into the bloodstream secondary to cardiomyocyte injury shows the potential of a specific cardiac biomarker that may have guiding properties for diagnosis, prognosis, and therapy. MyBP-C has been reported in many studies as a novel biomarker in myocardial infarction, which may help to quantify cardiomyocytes injury even more accurately than hs-TnI [35,36]. Also, due to its role in cardiac contraction and relaxation, dephosphorylation and subsequent release into the circulation was observed in patients with heart failure [37,38,39,40,41,42]. Furthermore, MyBP-C has emerged as a potential therapeutic target for the treatment of heart failure. Manipulating the phosphorylation status or expression levels of MyBP-C holds promise for altering cardiac contractility and improving cardiac function in HF patients [43].

Given its high cardiac specificity and its capacity to directly assess the cardiac function, MyBP-C is a promising biomarker in patients with AHF. The aim of our study was to investigate the clinical utility of this novel serum marker for diagnosis and short-term prognosis in subjects with AHF.

## 2. Materials and Methods

### 2.1. Study Design and Population

We conducted a prospective study that included a total of 49 subjects admitted in the Cardiology Clinic of “St. Spiridon” Emergency County Hospital (Iaşi, Romania), from February 2022 to September 2022. The AHF group included 27 subjects, while the control group consisted of 22 sex- and age-matched participants, either without heart failure (10 participants) or with stable, compensated HF (12 participants). For patients presenting with acute dyspnea, the final diagnosis of AHF was established for patients that met the Framingham criteria [44]. The major criteria were: acute pulmonary edema on X-ray, cardiomegaly (cardiothoracic index > 0.5 on X-ray), paroxysmal nocturnal dyspnea, orthopnea, jugular vein distension, hepatojugular reflux, pulmonary rales, the presence of the third heart sound (gallop rhythm), and weight loss > 4.5 kg in 5 days in response to treatment. The minor criteria were: ankle edema, dyspnea on exertion, hepatomegaly, nocturnal cough, pleural effusion, and tachycardia (heart rate > 120/min). For the diagnosis, at least 2 major criteria or 1 major criterion plus 2 minor criteria were required. The exclusion criteria for patients in both groups were: age < 18 years, presence of pregnancy, and presence of end-stage malignancies.

The study protocol was approved by the Ethics Committee of the “Grigore T. Popa” University of Medicine and Pharmacy Iaşi and by the Ethics Committee of the “St. Spiridon” Emergency Clinical Hospital in Iaşi. All research was conducted according to the ethical guidelines of the Declaration of Helsinki Principles, as revised in 2013. All patients signed a standard written informed consent form to participate in this study.

### 2.2. Study Procedures and Measurements

At the time of study entry, detailed clinical data were obtained from patients’ personal medical files. Data were completed with standard laboratory parameters: hemoglobin, hematocrit, serum iron, ferritin, sodium, renal function (urea, creatinine, serum bicarbonate, estimated glomerular filtration rate (eGFR)), glycemia, liver function (aspartate transaminase (AST), alanine transaminase (ALT), lactate dehydrogenase (LDH), gamma-glutamyl transpherase (GGT), alkaline phosphatase (ALP), total bilirubin, albumin), C-reactive protein, spot urinary sodium concentration (UNa+), urine albumin to creatinine ratio (ACR), uric acid, thyroid stimulating hormone (TSH), and creatine kinase myocardial band (CK-MB).

Standard biomarkers, NT-proBNP and hs-cTnI, used in the diagnosis and prognosis of HF were measured at admission in all patients, in EDTA plasma samples, using a PATHFASTTM Immunoanalyser. The PATHFAST NT-proBNP and hs-cTnI assay principle is based on the chemiluminescence enzyme immune assay (CLEIA) and *MAGTRATION^®^ methodology. The manufacturer reference interval is <15–128.3 pg/mL for the NT-proBNP and 0–29 ng/L for hs-cTnI, with the 99th percentile of URL of 29.7 ng/L for males and 20.3 ng/L for females.

For the quantification of MyBP-C concentration, blood samples were collected within 72 h after hospitalization in tubes containing potassium EDTA. Samples were immediately centrifuged (15 min at 1000× *g* at 2–8 °C within 30 min of collection) and kept frozen at −80 °C until their analysis in a single batch. We measured Cardiac Myosine Binding Protein C using a sandwich enzyme immunoassay (ELISA) kit (MYBPC3 ELISA Kit, Antibodies online GmbH, Aachen, Germany). The detection range of the kit was 0.15 ng/L–10 ng/L, with a minimum detection limit of 0.15 ng/L and a sensitivity of 0.062 ng/L.

Echocardiography was performed using a General ElectricVividTM V7 ultrasound device (General Electric, Boston, CA, USA) to evaluate cardiac parameters such as: left ventricle end-diastolic diameter (LVEDD), left ventricle end-systolic diameter (LVESD), left ventricle ejection fraction (LVEF), left atrium diameter (LAD), left atrium area (LAA), mitral annular plane systolic excursion (MAPSE), diastolic dysfunction and LV filling pressure by E/A, E/e′ ratio, right ventricle diameter (RVD), tricuspid annular plane systolic excursion (TAPSE), estimated systolic pulmonary arterial pressure (sPAP), mitral regurgitation (MR), and tricuspid regurgitation (TR).

Pulmonary congestion was assessed by pulmonary ultrasonography (LUS) at the end of standard two-dimensional echocardiography with the patient in the supine position. Using the same probe as was used for the echocardiographic study, we obtained LUS scans of four sites in each hemithorax, with the transducer position parallel to the ribs, at an imaging depth of 10–14 cm. Lung congestion was defined by the presence of B-lines; each site scored from 0 (A-lines) to 10 (white lung for coalescing B-lines). A thoracic area was considered positive if ≥2 B-lines were observed [45,46]. All LUS exams were performed by an operator unaware of laboratory data.

For the evaluation of prognosis, the clinical endpoints of the study were in-hospital mortality, mortality within 30 days, inability to undertake self-care and rehospitalization within 30 days.

### 2.3. Statistical Analysis

Categorical variables are expressed as number (percentage) and continuous variables as mean (SD) or median (interquartile range (IQR)). The comparisons between groups were assessed using the Student *t*-test, 2 test, Mann–Whitney U-test, Wilcoxon test, and Kruskal–Wallis test, as appropriate. For cardiac biomarkers (MyBP-C, NT-proBNP, hs-TnI), which had non-normally distributed values, medians and non-parametric tests (Mann–Whitney U-test, Kruskal–Wallis testing) were used.

The assessment of correlations between MyBP-C and clinical and biochemical variables was performed using Spearman’s rank correlation. Clinical characteristics between those who survived to 30 days from presentation versus those who died were compared using chi-square tests for categorical data and the Wilcoxon rank-sum test for continuous data.

To evaluate the utility of MyBP-C compared to NT-proBNP and hs-TnI for the diagnosis of AHF in subjects with dyspnea, as well as for identifying risk of death by 30 days in those with a diagnosis of AHF, we used receiver operating characteristic (ROC) curves, as recommended by DeLong et al. [47]. Optimal cut-off points for identifying and excluding the diagnosis of AHF, risk of 30-day mortality or 30-day recurrent HF event were obtained for MyBP-C. For the comparison of mortality risk within the two subgroups (with serum levels above and below the MyBP-C’s high-risk cut-off value), we performed Kaplan–Meier analysis for survival and used log-rank values to assess statistical significance.

We used multivariable logistic regression to identify the cardiac biomarkers that are predictive of death or death/rehospitalization at 30 days in all subjects. Goodness of fit was verified using the Hosmer–Lemeshow test. For each independent predictor in multivariable analyses, odds ratios (OR) were calculated with 95% confidence intervals (CI). For all statistical analyses, all *p* values were 2-sided, with results <0.05 considered. All statistical analyses were performed using SPSS/PC version 17.0 (SPSS) and Med-Calc v.22.018 (MedCalc Software).

## 3. Results

### 3.1. Baseline Characteristics

The study included 49 subjects: 27 patients diagnosed with AHF and 22 subjects in the control group (10 without HF and 12 with compensated HF). Among the AHF group, 44.4% were females and 55.6% were males. The average age (in years) of patients with AHF was 71.48 ± 9.86, while the mean age of the control group being 61.64 ± 13.08. The difference in age between the two groups may be due to the fact that older age is more often associated with AHF. There was no significant difference between the two groups regarding gender, BMI or presence of obesity (defined as BMI > 30 kg/m^2^) (*p* > 0.05). Also, habits such as smoking and alcohol abuse were equally present in the two groups (Table 1). Among the clinical parameters identified at admission, as expected, in the AHF group, the values were significantly higher for systolic blood pressure (SBP), respiratory rate and oxygen saturation (*p* < 0.05). Lung congestion assessed by LUS was also more prevalent in the AHF group (*p* < 0.001). Adverse prognostic parameters such as in-hospital mortality, 30-day mortality, inability to undertake self-care, and 30-day rehospitalization were all more frequent in AHF patients (*p* < 0.05). Most comorbidities had a similar frequency between groups, although hypertension, atrial fibrillation and CKD were significantly more present in patients with AHF (*p* < 0.05) (Table 1).

Laboratory tests complementary to cardiac biomarkers play a crucial role in estimating prognosis, exploring etiology, and guiding the treatment. Hemodynamic changes and neurohormonal activation in AHF lead to impaired kidney function, which also has an impact on prognosis. In our study, markers of renal dysfunction such as serum creatinine, ACR, UNa+, serum bicarbonate and eGFR had significantly modified values in patients with AHF (*p* < 0.05) (Table 2). Anemia and functional iron deficiency are frequently associated with HF and are considered negative prognostic factors. Our results show comparable levels for hemoglobin, hematocrit and ferritin between the groups (*p* > 0.005), but significantly lower values for iron in the AHF group (*p* = 0.015) (Table 2). Elevated levels were also observed for uric acid and total bilirubin in AHF patients, while tests such as those for C-reactive protein, albumin, sodium, and liver enzymes gave similar values between groups (Table 2).

Echocardiography is one of the main modes of investigation in AHF patients, providing vital insights into diagnosis and prognosis. Left ventricle dimensions, reduced ejection fraction, increased intracardiac pressures and pulmonary hypertension are markers of disease severity in HF, and predispose one to decompensation. Our data show significant differences between the AHF group and the control group for LVEDD, LVESD, LVEF, LAD, LAA, sPAP, MAPSE, moderate/severe MR and TR, E/e′ >15 (*p* < 0.05) (Table 3).

Stratification of HF according to etiology among the studied patients revealed that 55.6% (15 patients) had ischemic disease, 14.8% had alcoholic-dilated cardiomyopathy (4 patients), 18.5% had valvular disease (5 patients) and 11.1% had hypertensive heart disease (3 patients) (Table 4). Regarding New York Heart Association (NYHA) classification, class III was the most prevalent (21 patients, 42.9%), followed by class II (13 patients, 26.5%) and class IV (5 patients, 10.2%). No patient with HF had been classified as NYHA Class I (0 patients, 0%). Most of the patients with HF included in the study had reduced EF (88.9%). Acute pulmonary edema was the most frequent clinical presentation of AHF, at 63% (17 patients), followed by acute decompensated HF with 25.9% (7 patients). Cardiogenic shock was recorded in only 11.1% (three patients) (Table 4).

### 3.2. Profile of Cardiac Biomarkers

In line with the current guidelines, common HF biomarkers NT-proBNP and hs-TnI had higher values in patients with AHF (15783 vs. 89.90 pg/mL, *p* < 0.001, 349 vs. 3.2 ng/L, *p* < 0.001, respectively) (Table 2). For the new biomarker, MyBP-C, the results also showed increased plasma levels in patients with AHF (median = 54.88 ng/L, IQR = 0.01–542.70) compared to controls (median = 0.01 ng/L, IQR = 0.01–17.95) (*p* < 0.001) (Table 2).

In relation to factors precipitating AHF, MyBP-C did not vary significantly (*p* = 0.540), although lower values were recorded in patients with hypertension emergency. Similarly, NT-proBNP values were constant across different etiologies for decompensation (*p* = 0.456). hs-TnI, as a diagnostic biomarker of acute myocardial infarction, was the only biomarker showing significant level variations (Table 5).

Comparing EF in patients admitted with AHF, we found that all cardiac biomarkers had constant plasma concentrations among groups, with no significant differences between patients with reduced and preserved EF (Table 6).

Considering the different prognoses in various clinical presentations of AHF, we compared the median values of cardiac biomarkers among different types of AHF. All biomarkers had significantly higher values in cardiogenic shock (*p* < 0.05). Notably, the median plasma level of cMyBP-C was significantly higher in cardiogenic shock (513.30 ng/L) than in acute decompensated HF (56.84 ng/L) and acute pulmonary edema (35.76 ng/L), with a *p*-value < 0.001 (Table 7).

The NYHA classification also serves as a tool to assess disease burden and prognosis. In this regard, we compared the biomarker levels in different NYHA classes. MyBP-C showed similar results to the usual cardiac biomarkers, NT-proBNP and hs-TnI. The median plasma levels of MyBP-C were significantly higher in class IV NYHA (97.33 ng/L) than in class III (27.04 ng/L) and class II (0.01 ng/L), with *p* = 0.011 (Table 8). Table 8 also shows that, compared with NT-proBNP and hs-TnI, there was no significant difference between the plasma levels of MyBP-C in the mildest NYHA class patients (class II) and the control group (*p* > 0.05).

Comparing the median biomarkers’ concentrations in ischemic versus non-ischemic origin of AHF, MyBP-C had more elevated values in patients with AMI (*p* = 0.046), which supports its role in acute ischemia (Table 9). Similarly, the biomarker already established in the diagnosis of AMI, hs-TnI, had higher concentrations in this group (*p* < 0.001, Table 9). Meanwhile, there were no significant differences in median concentrations for NT-proBNP (Table 9).

Looking at specific correlations between MyBP-C and certain relevant parameters commonly assessed in patients with AHF, we identified positive correlations with clinical aspects suggestive of AHF (dyspnea, pulmonary rales, lung congestion) and a negative correlation with oxygen saturation (r = −0.487, *p* < 0.001). Age, gender, smoking and alcohol abuse were not influencing factors for MyBP-C levels (*p* > 0.05). Also, MyBP-C concentration was not associated with obesity, systolic blood pressure (SBP), diastolic blood pressure (DBP), or heart rate (HR) (*p* > 0.005).

Among the comorbidities present in patients with AHF, we note than only AMI (r = 0.535, *p* <0.001) and atrial fibrillation (r = 0.456; *p* = 0.001) influenced MyBP-C values, while diseases like pulmonary embolism (PE), CKD, COPD, anemia, sepsis and infection were not associated with MyBP-C levels (*p* > 0.05) (Table 10). In contrast, NT-proBNP values were modified by AMI, PE, sepsis, CKD, COPD and hs-TnI levels by the presence of AMI, PE, atrial fibrillation and COPD (Table 10).

Poor outcome predictors, such as cardiogenic shock, inotropic support, loop diuretic dose, invasive ventilation and length of hospital stay, had strong positive correlations with My-BPC. Also, its predictive value is supported further by its association with in-hospital mortality, 30-day mortality and 30-day rehospitalization rate (Table 10).

Among the laboratory tests with prognostic value, C-reactive protein, serum creatinine, eGFR, sodium, UNa^+^, urinary ACR, albumin, uric acid, AST and serum bicarbonate had strong correlations with MyBP-C concentrations (Table 11).

Concerning echocardiography parameters, we observed that the levels of this novel biomarker varied according to left chamber dimensions, LVEF, LV systolic dysfunction, sPAP, MAPSE, elevated LV filling pressures (E/e′ > 15) and IVC. Higher values of MyBP-C were associated with the echocardiographic criteria of HF severity (Table 11).

Both NT-proBNP (r = 0.727, *p* < 0.001) and hs-TnI (r = 0.604, *p* = < 0.001) had a positive correlation with MyBP-C (Table 12).

### 3.3. Diagnostic Performance of MyBP-C

**Figure 1:**
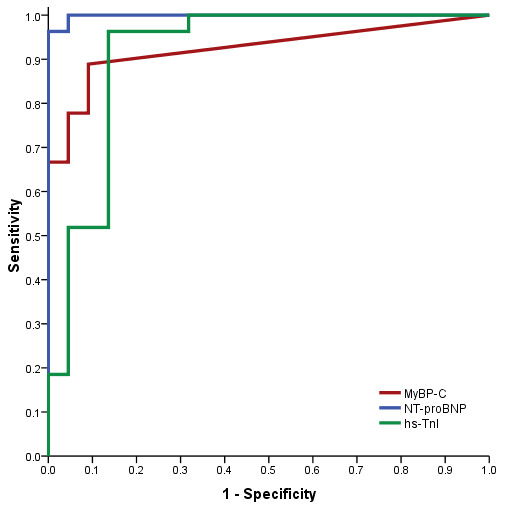
Combined receiver operating characteristic (ROC) curves for NT-proBNP, MyBP-C and hs-TnI for the diagnosis of AHF in dyspneic patients.

To assess the diagnostic performance of MyBP-C, we performed ROC analysis, which showed an area under the curve (AUC) of 0.92 (*p* < 0.001), with an optimal cut-off of 4.61 ng/L yielding a sensitivity of 85% and a specificity of 90%, these being results that support the potential role of MyBP-C in predicting AHF. For the currently recommended cut-off for NT-proBNP of 300 pg/mL, the sensitivity was 100% and the specificity was 77%. NT-proBNP had the greatest AUC at 0.998. The ROC analysis for hs-TnI showed an AUC of 0.91 (*p* < 0.001), with a sensitivity of 81% and a specificity of 86% for a cut-off value of 29 ng/L. A comparison of the three ROC curves is shown in Figure 1 (Table 13).

### 3.4. Prognostic Value of MyBP-C

Among the 27 subjects with AHF, 11 (40.7%) had recurrent HF within 30 days, whereas 9 patients (33.3%) died by 30-day follow-up. Demographic characteristics expressed as a function of 30-day survival are shown in Table 14. Mortality within 30 days was associated with the presence of atrial fibrillation, higher levels of uric acid, lower values for serum bicarbonate and albumin, and dilated left atrium (Table 14).

**Figure 2:**
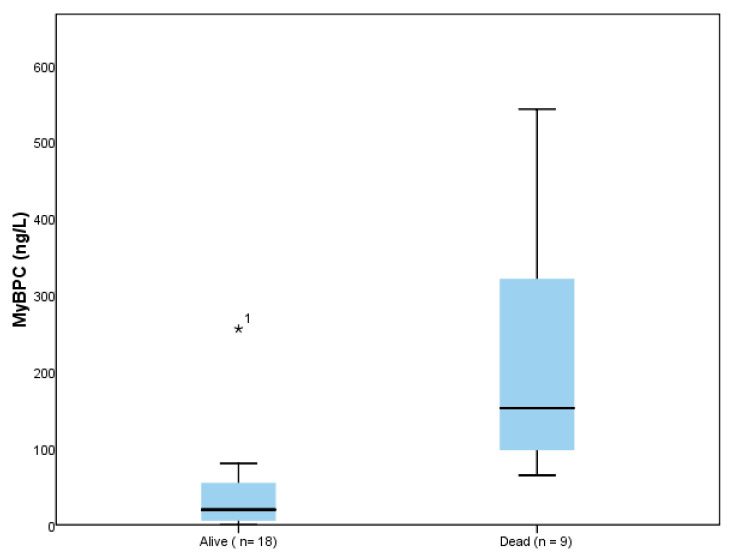
Median MyBP-C levels among HF patients who died (*n* = 9) within 30 days and those who survived (*n* = 18). Boxes = interquartile ranges; whiskers = 5th and 95th percentiles.

Median concentrations of MyBP-C were significantly higher among those subjects dying within 30 days of follow-up (152.44 ng/L, IQR = 81.05–417.26 ng/L) than in those surviving (19.73 ng/L, IQR = 4.92–55.37 ng/L, *p* < 0.001) (Figure 2). In comparison with MyBP-C, NT-proBNP and hs-TnI had similar values among the two groups (Table 14).

**Figure 3:**
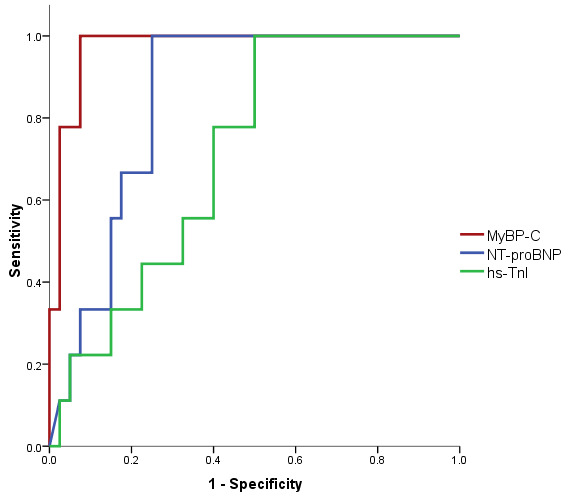
ROC curve for the relationship between cardiac biomarkers and total mortality rate within 30 days.

The ROC analysis for mortality within 30 days showed an AUC for MyBP-C of 0.972 (*p* < 0.001), an AUC for NT-proBNP of 0.849 (*p* = 0.001), and an AUC for hs-TnI of 0.714 (*p* = 0.078), as depicted in Figure 3. In contrast to the diagnostic ability in AHF, where NT-proBNP was superior to MyBP-C, MyBP-C showed a significantly greater AUC than NT-proBNP (*p* = 0.033) and hs-TnI (*p* = 0.001) in patients who died within 30 days (Table 15). The optimal cut-off point for MyBP-C for prediction of 30 days mortality was 64.69 ng/L, which is 88.9% sensitive and 92.5% specific.

**Figure 4:**
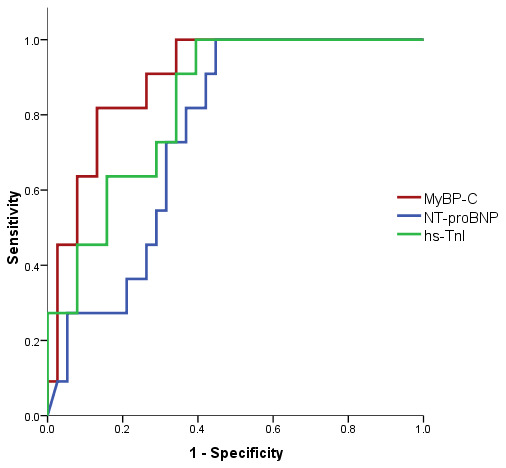
ROC curve for the relationship between cardiac biomarkers and rehospitalization rate within 30 days.

Similar results were obtained for rehospitalization within 30 days, with superior AUC values for MyBP-C (AUC = 0.897, *p* < 0.001) when compared with NT-proBNP (AUC = 0.750, *p* = 0.012) and hs-TnI (AUC = 0.833, *p* = 0.001) (Figure 4, Table 16). The optimal cut-off point for MyBP-C for prediction of rehospitalization within 30 days was 47.46 ng/L, with a sensitivity of 81.8% and specificity of 86.8%.

**Figure 5:**
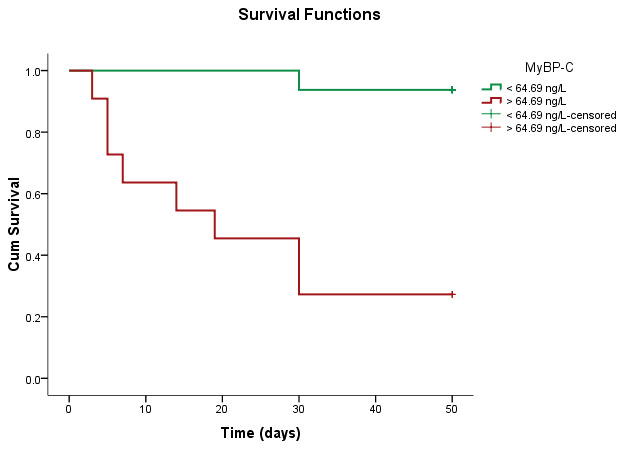
Kaplan–Meier survival curves according to the high-risk cut-off.

By plotting the Kaplan–Meier curves according to MyBP-C concentration, we observed diminished survivability in patients with MyBP-C values higher than 64.69 ng/L (Figure 5). The log-rank test confirmed a statistically significant difference between the two subgroups, according to the specified 64.69 ng/L cut-off value (Table 17).

In adjusted multivariate analysis, MyBP-C levels were the best predictor of mortality within 30 days (OR 1.08, 95% CI 1 to 1.16, *p* = 0.039; Table 13), superior to NT-proBNP or hs-TnI for this purpose. A second adjusted multivariate analysis was performed, examining the combined end point of death and recurrent HF within 30 days. The analysis confirmed that MyBP-C levels were the best predictor of events (OR 1.12, 95% CI 1.02 to 1.22, *p* = 0.014) when compared to the other cardiac biomarkers (NT-proBNP and hs-TnI) (Table 18).

## 4. Discussion

AHF is a condition associated with high mortality that requires prompt diagnosis and intervention [6]. Biomarkers stand out as excellent diagnostic tools, due to their multiple advantages: accessibility, feasibility, reproducibility, predictive value capacity and non-invasive nature. One of the main strengths of biomarkers is their ability to reflect different pathological mechanisms, which makes them perfect tools in conditions involving complex pathophysiological processes, such as cardiovascular diseases. Also, by developing multimarker strategies, the combination of different biomarkers can provide a comprehensive diagnostic and prognostic assessment [8].

Pathophysiological pathways in AHF involve renin–angiotensin–aldosterone and sympathetic nervous system activation, sodium and water retention, arterial and venous vasoconstriction, cardiac fibrosis, and remodeling. Cardiac remodeling is a consequence of cardiac myocyte injury and death, extracellular matrix changes, and inflammatory reactions, which over time cause irreversible damage [6]. Moreover, pathological processes are accentuated by low cardiac output and blood pressure, which further reduce coronary circulation and oxygen delivery to the heart muscle. NPs are released into circulation secondary to elevated filling pressures and the stretching of the ventricle walls [48]. These are the gold standard biomarkers recommended for ruling out the diagnosis of AHF according to current guidelines [3]. They have a high sensitivity for the diagnosis of AHF, but a low specificity, as their values are influenced by several cardiac and non-cardiac conditions that alter hemodynamic balance, fluid status and cardiac filling pressures: age, obesity, atrial fibrillation, anemia, sepsis, CKD and pulmonary hypertension [49,50].

The compromise in blood supply is followed by cardiac injury and cardiac cell apoptosis, leading to the release of intracellular contents like cardiac troponin [19,51]. In AHF, the role of troponin is to diagnose AMI and to provide prognostic information [3,52]. Troponins have a low specificity for AHF diagnosis, with elevated levels recorded in myocarditis, pulmonary embolism and acute coronary syndromes [19]. However, they have an important role in AHF patients, where elevated levels were found to be associated with increased mortality and morbidity [52].

Cardiac myocyte injury activates an inflammatory reaction and increases oxidative stress, with the release of cytokines, adhesion molecules and chemokines. ST2, GDF-15 and galectin-3 are markers of inflammation and fibrosis. ST2 is part of the interleukin-1 receptor family, released into circulation secondary to myocardial stretch and injury, inflammation and fibrosis. ST2 levels were associated with worse outcomes in AHF patients, which established its use as a prognostic biomarker [53,54,55,56]. Also with prognosis properties, GDF-15 and galectin-3 are released in response to cardiac injury and inflammation, promoting the activation and proliferation of cardiac fibroblasts and the deposition of extracellular matrix (ECM) proteins [57,58,59,60,61,62,63]. Matrix metalloproteinases (MMPs) are molecules involved in the degradation of ECM, such as collagen, elastin, and proteoglycans [64,65]. In AHF, in the context of hemodynamic stress, neurohormonal activation, and inflammatory cytokines, the levels of MMPs are elevated, mediating ECM remodeling, inflammation, fibrosis, and ventricular remodeling processes [66,67,68]. MMPs are complex molecules that show multiple uses in HF. Higher plasma MMP levels correlated with congestive HF, regardless of etiology, and were associated with a worse prognosis through an increased risk of death or hospitalizations [69,70,71].

The utilization of biomarkers in the diagnosis and prognosis of HF has been a subject of extensive research [9,10,72], and the role of MyBP-C in this context is a significant area of interest [39,73]. This article brings new evidence related to the potential use of MyBP-C as a biomarker in HF, particularly in comparison to established markers like NT-proBNP and hs-TnI, and its relevance in differentiating HF from other causes of dyspnea. Combining the results in a multimarker approach can lead to a more complete profile of the patient’s disease. The aim of our study was to evaluate the role of MyBP-C in AHF as a useful biomarker for diagnosis and risk stratification.

MyBP-C is a cardiac-specific protein involved in heart contraction that can be released in increased amounts into the plasma as a result of cardiac injury [32,38,74]. Cardiac injury is the process of disruption of cardiac cell integrity, followed by the release of intracellular proteins, like MyBP-C, into the bloodstream. In AHF, myocyte injury is part of the pathophysiological pathway, through hemodynamic stress, inflammatory processes, neurohormonal activation, imbalance between myocardial oxygen supply and demand, and increased myocardial workload [6]. Compared to hs-TnI, also a marker of cardiomyocyte injury, MyBP-C showed a better accuracy for injury detection, via a more rapid release and clearance kinetics [36,75,76,77]. In this context, we analyzed the contribution of this biomarker to a more specific diagnosis of AHF.

Our study showed that MyBP-C exhibits significantly elevated plasma levels in patients with AHF compared to controls. Also, clinical features typically present in patients with AHF, such as dyspnea, pulmonary rales, low oxygen saturation and pulmonary congestion, were associated with higher levels of MyBP-C compared to controls. These results highlight the capacity of MyBP-C to identify patients with manifestations of HF decompensation. Importantly, MyBP-C values were not influenced by age, gender, smoking status, alcohol abuse, or obesity, which supports its use independently of clinical and demographic factors. In comparison, age was an influencing factor for both NT-proBNP and hs-TnI, consistent with previous studies showing reduced specificity in older patients [75,76].

The results of our ROC analysis indicate the robust discriminatory ability of MyBP-C in distinguishing patients with and without AHF, although slightly inferior to NT-proBNP. These results are in agreement with previous studies that focused on the diagnostic ability of MyBP-C. In 2017, Doaa El Amrousy et al. revealed in pediatric patients that plasma levels of MyBP-C were associated with AHF [39], the diagnostic ability of the biomarker in children being also confirmed in 2020 by Eman M. El-Moghazy et al., and in 2021 by Ahmed A Khatab et al. [40,77]. For adult patients, we found a single clinical study focused on the role of MyBP-C in AHF diagnosis, published by Nikola Kozhuharov et al. in 2021 [41]. The researchers conducted, in a prospective multicenter diagnostic study, a comparison between patients diagnosed with AHF and those presenting with other causes for acute dyspnea. They observed that plasma concentrations of MyBP-C were significantly higher in patients diagnosed with AHF. Similar to our study, they noted a high diagnostic accuracy of MyBP-C quantified by the AUC, but it was lower than NT-proBNP, which underlines the continuing significant role of NT-proBNP in AHF diagnosis.

In our study, MyBP-C showed positive correlations with established HF biomarkers (NT-proBNP and hs-TnI), which highlights its potential as an additional diagnostic tool in AHF, especially in cases where conventional biomarkers may not provide conclusive results. The relationship of MyBP-C with NT-proBNP and hs-TnI is supported by previous reports and is driven by the complex changes in AHF associated with cardiac injury, myocardial stretching, and cardiac pressure overload [39,40,41]. The diagnostic accuracy and prognostic value in AHF can be improved by a multimarker approach, which allows the evaluation of different pathophysiological pathways.

When examining MyBP-C levels in patients with AHF and different precipitating factors, the results were comparable, showing that the cause for HF decompensation is not related to MyBP-C variation. Among classical biomarkers, hs-TnI had significantly higher values in patients with acute coronary syndrome as a cause for cardiac decompensation, which is in agreement with current guidelines and previous studies recommending its use for the diagnosis of AMI [3,77,78]. Although they share a similar mechanism, being released secondary to cardiac injury, these results confirm the superior sensitivity of MyBP-C and its constant role in detecting AHF, independent of cardiac ischemia. Ejection fraction (EF) is an echocardiographic parameter that shows the degree of left ventricle systolic dysfunction, and it is being used currently as a tool for classification in HF [3]. In cases of preserved EF or mildly reduced EF, the diagnosis of AHF may be challenging, and focused more on biomarker results. NPs in particular are the preferred used biomarkers due to their ability to increase across all ranges of EF in HF [3,79]. In our research, MyBP-C exhibited constant values in AHF patients independent of EF type. As acute decompensation is a consequence of increased congestion, volume and pressure overload and myocardial stretch, both NT-proBNP and MyBP-C have the ability to also detect these changes in patients with preserved EF. These results suggest that MyBP-C is an effective biomarker that can be used in detecting AHF in all clinical scenarios.

The NYHA classification is commonly used in patients with chronic HF to better stratify prognosis and disease severity [3]. Higher NYHA class was associated with increased mortality and hospitalization in patients with HF, ranging from 10 to 15% mortality for NYHA class I and II to 50% for class IV patients [80,81,82]. The results of our study show significantly increased values for all cardiac biomarkers in patients classified in class IV NYHA. Both NT-proBNP and cardiac troponin have been correlated in other studies with the NYHA functional class of heart failure, which also established them as prognostic markers [83,84,85]. MyBP-C confirms in this context a dynamic influenced by increasing disease severity and congestion.

The results in the scientific literature concerning the identification of an optimal cut-off point for MyBP-C in AHF diagnosis are conflicted. For pediatric patients, the cut-off point varied at 45 ng/mL [39], 70 ng/mL [77] or 63.3 ng/mL [40]. In the adult population, Nikola Kozhuharov et al. achieved in their study a sensitivity of 95% at a cut-off concentration of 16 ng/L, with a lower specificity (37% vs. 55%) compared to NT-proBNP at a cut-off point of 300 pg/mL [41]. In contrast, the cut-off value in our study was set at 4.61 ng/L, with a sensitivity of 85% and a specificity of 90%. Its higher specificity compared to NT-proBNP (90% vs. 77%) suggests MyBP-C may offer better discrimination between true positive and false positive cases, and its potential use as a rule-in diagnostic biomarker. Also, in our study, MyBP-C demonstrated a comparable diagnostic performance to hs-TnI, which may be due to the similar biomarker release mechanisms involving cardiac injury.

The findings support the growing body of evidence focused on MyBP-C utility in AHF [39,40,41,73]. Its high specificity makes it particularly valuable in ruling in AHF cases, potentially reducing unnecessary diagnostic procedures and improving resource allocation in clinical settings. While NT-proBNP remains the gold standard biomarker for HF diagnosis, the addition of MyBP-C provides complementary information and may improve overall diagnostic accuracy, especially in cases where individual biomarkers may yield inconclusive or discordant results. MyBP-C’s ability to capture different aspects of HF pathophysiology, such as myocardial damage and dysfunction, complements the neurohormonal activation reflected by NT-proBNP. Integrating MyBP-C into routine clinical practice may thus contribute to the more precise and timely diagnosis of AHF, ultimately leading to improved patient outcomes and healthcare efficiency.

Clinical and paraclinical parameters often interfere with cardiac biomarkers in AHF, which influences the interpretation of their results. NT-proBNP levels in particular are also elevated in exacerbations of COPD, CKD, anemia, atrial fibrillation and pulmonary hypertension, diminishing its ability to distinguish the exact cause of acute dyspnea [14,86,87,88,89,90]. In our study NT-proBNP levels were higher in patients with AMI, PE, sepsis, atrial fibrillation, CKD and COPD, conditions that decrease NPs specificity, as they are characterized by increased cardiac stress, volume overload, myocardial injury or impaired clearance. These mechanisms may also lead to increased cardiac troponin levels, as shown in our study. In contrast, comorbidities such as diabetes mellitus, CKD, PE, sepsis and COPD were not found to be associated with MyBP-C levels in our results, highlighting the additional potential role of this novel biomarker in these clinical situations. The presence of atrial fibrillation was correlated with higher levels for all biomarkers studied, possibly due to increased atrial wall stress, increased cardiac pressure, and myocardial injury. As the association of atrial fibrillation with NT-proBNP and hs-TnI levels was previously described, further research is needed to elucidate the specific mechanisms underlying MyBP-C release in AF and its potential implications for atrial remodeling and function [91,92,93].

AMI is a critical concern in the setting of AHF, with a significant impact on mortality, long-term survival, and life quality. Patients presenting with AHF associated with AMI typically exhibit more severe clinical symptoms, higher rates of comorbid conditions, and greater impairment of left ventricular function. These factors collectively contribute to the poorer prognosis observed in this patient population [1,94]. MyBP-C has been identified as a potential biomarker for early AMI detection, due to its role as a sarcomeric protein, crucial for cardiac muscle contraction regulation. During ischemic injury, MyBP-C is sensitive to dephosphorylation and undergoes proteolytic degradation, which leads to its release into the circulation [32,95,96]. Elevated levels of MyBP-C and its fragments have been observed in post-MI samples from both rats and humans, highlighting its potential use as a novel biomarker for accurately diagnosing MI [95,96]. Our findings support previous research efforts. We observed a significant elevation in MyBP-C levels among patients with AHF associated with AMI, similar to hs-TnI, a well-established biomarker for AMI diagnosis. In comparison, NT-proBNP concentration did not differ significantly between ischemic and non-ischemic HF patients. While NT-proBNP is a widely used biomarker for HF diagnosis and prognosis, MyBP-C and hs-TnI may offer additional diagnostic value, particularly in identifying patients with AMI within the HF population.

MyBP-C’s role in identifying high-risk patients who require immediate intervention and intensive management was further investigated by comparing the biomarker levels among different types of AHF presentations. In our study, MyBP-C levels were significantly elevated in patients with cardiogenic shock, similarly to NT-proBNP and hs-TnI. Due to severe tissue congestion and hypoperfusion, cardiogenic shock is associated with increased cardiac biomarkers and worse outcomes in AHF [97,98].

Also, we observed higher plasma concentrations in patients with echocardiography parameters indicative of HF severity, including left chambers dimensions, left ventricle ejection fraction, sPAP and MAPSE, moderate/severe MR, LV systolic dysfunction, and increased LV filling pressures. These results suggest MyBP-C may contribute to the assessment of HF severity and may aid clinicians in tailoring treatment strategies based on individual patient characteristics. The ability of MyBP-C to differentiate more severe forms of HF has also been proven in previous studies, highlighting its potential as a marker of adverse cardiac remodeling and dysfunction [34,39].

Incorporating MyBP-C measurements into risk stratification algorithms could improve prognostic accuracy and guide therapeutic decision-making in the management of AHF. Various laboratory tests with prognostic value, such as C-reactive protein, serum creatinine, sodium, urinary sodium, urinary albumin–creatinine ratio, albumin, uric acid, AST, and serum bicarbonate, showed strong correlations with MyBP-C levels and standard cardiac biomarkers (NT-proBNP, hs-TnI). Renal dysfunction is a common association in patients with AHF as part of the cardiorenal syndrome, leading to a worse prognosis [99]. Through reduced clearance, neurohormonal activation, volume overload and myocardial injury, kidney failure is associated with elevated levels of cardiac biomarkers [100,101,102]. Our results reveal similar findings for MyBP-C in this context, supporting its parallel trajectory with markers of negative outcomes.

Its prognostic significance is also evidenced by direct associations with predictors of poor outcomes, such as the need for inotropic support or invasive ventilation, in-hospital mortality, length of hospital stay, and 30-day readmission. Elevated levels of MyBP-C were significantly associated with mortality and recurrent HF within 30 days. Furthermore, the superior diagnostic accuracy of MyBP-C compared to NT-proBNP and hs-TnI in predicting these outcomes highlights its clinical relevance in risk stratification and the early identification of high-risk patients. These findings are in accordance with previous work on the clinical use of MyBP-C. In their paper, Nikola Kozhuharov et al. particularly focused on long-term outcomes (all-cause mortality and AHF rehospitalization within 360 days), showing that MyBP-C has incremental prognostic value over NT-proBNP and hs-TnI [41].

The identification of an optimal cut-off point for prognostic purposes provides clinicians with a valuable tool for risk stratification and early intervention in patients with AHF. In our study, we identified higher mortality rates at a cut-off value of 64.69 ng/L for MyBP-C, comparable to the study of Nikola Kozhuharov et al. [41].

### Limitations of the Study

The results must be interpreted while taking into account several of the limitations of our study. The small sample size may limit the generalizability of the results. Also, the isolated measurement of MyBP-C levels at presentation limits the clinical applicability. Future analyses in additional prospective studies are needed to confirm the diagnostic and prognostic value of MyBP-C in larger and more diverse patient populations. Longitudinal studies may provide valuable clinical insights by tracking MyBP-C levels over time and evaluating its response to therapeutic interventions. Additionally, further research may elucidate the optimal cut-off values and clinical thresholds for MyBP-C in differentiating AHF and predicting outcomes.

## 5. Conclusions

The study brings new evidence supporting the role of MyBP-C as a comprehensive biomarker in AHF. While NT-proBNP remains the gold standard biomarker in AHF, MyBP-C has been shown to have an impressive diagnostic performance with high sensitivity and specificity, providing complementary information. MyBP-C may serve as a valuable adjunctive tool in enhancing diagnostic accuracy and guiding clinical decisions in AHF.

In addition, MyBP-C has a promising role as a prognostic biomarker in AHF, being able to complement existing markers, and thus achieve better risk stratification and the prediction of short-term outcomes.

Further research efforts are warranted to validate these findings and translate MyBP-C into routine clinical practice for improved patient care and management.

## Figures and Tables

**Table 1:** Baseline characteristics.

Characteristics	Total (*n* = 49)	AHF *(n* = 27)	Control Group			*p*-Value
			Total Control (*n* = 22)	No HF *(n* = 10)	Compensated HF (*n* = 12)	
Gender						0.567
Male, *n* (%)	29 (59.2%)	15 (55.6%)	14 (63.6%)	7 (70%)	7(58.3%)	
Female, *n* (%)	20 (40.8%)	12 (44.4%)	8 (36.4%)	3 (30%)	5 (41.7%)	
Age, y (mean ± SD)	67.06 ± 12.33	71.48 ± 9.86	61.64 ± 13.08	59.60 ± 13.83	63.33 ± 12.78	0.004
Smoking, *n* (%)	29 (59.2%)	17 (63%)	12 (54.5%)	6 (60%)	6 (50%)	0.551
Alcohol abuse, *n* (%)	13 (29.5%)	8 (29.6%)	5 (22.7%)	2 (20%)	3 (25%)	0.586
Clinical Parameters						
BMI, kg/m^2^ (mean ± SD)	28.98 ± 5.35	28.73 ± 5.66	29.30 ± 5.06	29.50 ± 5.41	29.13 ± 5.00	0.712
Obesity, *n* (%)	22 (44.9%)	11 (40.7%)	11 (50%)	5 (50%)	6 (50%)	0.517
SBP, mmHg (mean ± SD)	149.51 ± 37.31	160.26 ± 39.68	136.32 ± 30.02	125.10 ± 22.80	145.67 ± 32.95	0.024
DBP, mmHg (mean ± SD)	88.94 ± 21.71	91.93 ± 25.37	85.27 ± 15.97	76.00 ± 9.53	93.00 ± 16.41	0.291
Heart rate, bpm (mean ± SD)	98.59 ± 24.40	103.48 ± 24.61	92.59 ± 23.30	82.00 ± 16.12	101.42 ± 25.24	0.121
Respiratory rate, breaths/min (mean ± SD)	17.90 ± 3.98	19.19 ± 4.38	16.32 ± 2.76	16.20 ± 1.68	16.42 ± 3.50	0.011
Oxygen saturation, % (mean ± SD)	91.35 ± 8.13	87.44 ± 9.14	96.14 ± 2.03	96.90 ± 1.59	95.50 ± 2.19	<0.001
Temperature, °C (mean ± SD)	36.29 ± 0.46	36.43 ± 0.54	36.11 ± 0.25	36.09 ± 0.26	36.13 ± 0.26	0.014
Lung congestion, *n* (%)	21 (42.9%)	20 (74.1%)	1 (4.5%)	0 (0%)	1 (8.3%)	<0.001
In-hospital mortality, *n* (%)	5 (10.2%)	5 (18.5%)	0 (0%)	0 (0%)	0 (0%)	0.033
Inability of self-care, *n* (%)	11 (22.4%)	10 (37%)	1 (4.5%)	0 (0%)	1 (8.3%)	0.007
Mortality within 30 days, *n* (%)	4 (8.2%)	4 (14.8%)	0 (0%)	0 (0%)	0 (0%)	0.060
Rehospitalization within 30 days, *n* (%)	11 (22.4%)	11 (40.7%)	0 (0%)	0 (0%)	0 (0%)	0.001
Medical History						
Hypertension, *n* (%)	35 (71.4%)	23 (85.2%)	12 (54.5%)	5 (50%)	7 (58.3%)	0.018
Diabetes mellitus, *n* (%)	20 (40.8%)	13 (48.1%)	7 (31.8%)	2 (20%)	5 (41.7%)	0.247
Dyslipidemia, *n* (%)	20 (40.8%)	13 (48.1%)	7 (31.8%)	3 (30%)	4 (33.3%)	0.247
CAD, *n* (%)	20 (40.8%)	13 (48.1%)	7 (31.8%)	1 (10%)	6 (50%)	0.247
Atrial fibrillation, *n* (%)	21 (42.9%)	17 (63%)	4 (18.2%)	0 (0%)	4 (33.3%)	0.002
CKD, *n* (%)	38 (79.2%)	24 (88.9%)	14 (66.7%)	5 (55.6%)	9 (75%)	0.060
Stroke, *n* (%)	3 (6.1%)	3 (11.1%)	0 (0%)	0 (0%)	0 (0%)	0.107
PAD, *n* (%)	2 (4.1%)	2 (7.4%)	0 (0%)	0 (0%)	0 (0%)	0.192
COPD, *n* (%)	7 (14.3%)	6 (22.2%)	1 (4.5%)	0 (0%)	1 (8.3%)	0.079

Abbreviations: AHF—acute heart failure; HF—heart failure; *n*—number of patients; y—years; SD—standard deviation; SBP—systolic blood pressure; DBP—diastolic blood pressure; BMI—body mass index; bmp—beats per minute; CAD—coronary artery disease; CKD—chronic kidney disease; PAD—peripheral artery disease; COPD—chronic obstructive pulmonary disease.

**Table 2:** Laboratory parameters.

Parameter	Total (*n* = 49)	AHF (*n* = 27)	Control Group			*p*-Value
			Total Control *(n* = 22)	No HF (*n* = 10)	Compensated HF *(n* = 12)	
Hemoglobin, g/dL (mean ± SD)	13.13 ± 2.11	12.83 ± 2.44	13.5 ± 1.60	13.81 ± 1.78	13.25 ± 1.47	0.271
Hematocrit, % (mean ± SD)	39.74 ± 5.99	39.51 ± 7.06	40.02 ± 4.50	40.95 ± 4.90	39.25 ± 4.18	0.770
Serum iron, µg/dL (mean ± SD)	57.73 ± 37.19	46.44 ± 22.82	72.24 ± 46.68	62.78 ± 26.99	79.33 ± 57.49	0.015
Ferritin, ng/mL (mean ± SD)	243.39 ± 335.79	280.88 ± 419.11	190.47 ± 155.88	295.50 ± 169.82	133.18 ± 119.28	0.403
C-reactive protein, mg/dL (mean ± SD)	2.42 ± 2.64	2.94 ± 2.83	1.79 ± 2.28	2.29 ± 2.46	1.37 ± 2.14	0.130
Sodium, mmol/L (mean ± SD)	138 ± 5.39	136.78 ± 6.61	139.50 ± 2.84	140.20 ± 2.65	138.92 ± 2.96	0.079
Potassium, mmol/L (mean ± SD)	4.47 ± 0.54	4.58 ± 0.62	4.33 ± 0.38	4.22 ± 0.27	4.42 ± 0.43	0.104
Urea, mg/dL (mean ± SD)	57.86 ± 26.15	64.11 ± 28.39	50.18 ± 21.30	40.80 ± 19.01	58.00 ± 20.59	0.063
Serum creatinine, mg/dL (mean ± SD)	1.18 ± 0.52	1.33 ± 0.59	0.99 ± 0.34	0.90 ± 0.36	1.07 ± 0.33	0.023
ACR, mg/g (mean ± SD)	168.82 ± 371.98	288.27 ± 471.32	22.21 ± 19.46	10.80 ± 9.99	31.72 ± 20.60	0.011
UNa^+^, mmol/L (mean ± SD)	77.45 ± 47.61	60.22 ± 31.29	98.59 ± 55.85	117.50 ± 52.56	82.83 ± 55.67	0.004
Serum bicarbonate, mEq/L (mean ± SD)	23.89 ± 4.46	22.58 ± 4.16	25.65 ± 4.35	24.67 ± 3.62	26.35 ± 4.87	0.030
eGFR, ml/min/1,73 m^2^ (mean ± SD)	65.92 ± 25.95	55.30 ± 21.79	78.95 ± 25.05	87.60 ± 20.23	71.75 ± 27.19	0.001
Albumin, mg/dL (mean ± SD)	57.65 ± 5.99	56.38 ± 6.64	59.20 ± 4.78	58.45 ± 3.81	59.84 ± 5.55	0.102
AST, IU/L (mean ± SD)	103.67 ± 377.02	161.78 ± 498.49	28.95 ± 24.11	21.67 ± 6.94	34.42 ± 30.76	0.230
ALT, IU/L (mean ± SD)	82.33 ± 283.86	124.44 ± 375.65	28.19 ± 20.913	22.56 ± 10.52	32.42 ± 25.86	0.248
GGT, IU/L (mean ± SD)	92.60 ± 135.05	125.67 ± 169.85	50.10 ± 44.54	46.89 ± 22.73	52.50 ± 56.71	0.530
Glycemia, mg/dL (mean ± SD)	157 ± 85.23	175.67 ± 94.70	131.80 ± 64.45	111.88 ± 29.04	145.08 ± 78.47	0.810
Uric acid, mg/dL (mean ± SD)	7.22 ± 2.53	8.05 ± 2.52	6.16 ± 2.15	5.81 ± 1.83	6.39 ± 2.39	0.010
Total bilirubin, mg/dL (mean ± SD)	1.01 ± 0.90	1.30 ± 1.12	0.66 ± 0.27	0.64 ± 0.31	0.67 ± 0.25	0.012
TSH, mIU/L (mean ± SD)	2.09 ± 1.83	2.25 ± 2.22	1.89 ± 1.25	1.89 ± 1.30	1.89 ± 1.26	0.501
CK-MB, mg/dL (mean ± SD)	50.97 ± 105.36	69.22 ± 138.59	28.57 ± 25.23	19.76 ± 10.35	35.91 ± 31.59	0.182
MyBP-C, ng/L (median, IQR)	5.23 (0.01–60.73)	54.88 (9.59–123.87)	0.01 (0.01–0.01)	0.01 (0.01–0.01)	0.01 (0.01–0.01)	<0.001
NT-proBNP, pg/mL (median, IQR)	3925 (105–15923)	15783 (7153–21113)	89.90 (53.75–305.75)	53.50 (39.73–73.55)	282.50 (102.50–385.75)	<0.001
hs-cTnI, ng/L (median,IQR)	42.80 (3.82–510)	349 (53–3885)	3.2 (2.08–7.66)	2.29 (1.33–2.74)	7.28 (3.66–176.10)	<0.001

Abbreviations: AHF—acute heart failure; HF—heart failure; *n*—number of patients; y—years; SD—standard deviation; IQR—interquartile range; ACR—albumin creatinin ratio; UNa+—urinary sodium; eGFR—estimated glomerular filtration rate; AST—aspartate transaminase; ALT—alanine transaminase; GGT—gamma-glutamyl transpherase; TSH—thyroid stimulating hormone; CK-MB—creatine kinase myocardial band; MyBP-C—myosin binding protein C; NT-proBNP—N-terminal pro b-type natriuretic peptide; hs-TnI—high sensitivity troponin I.

**Table 3:** Echocardiography parameters.

Parameter	Total (*n* = 49)	AHF (*n* = 27)	Control Group			*p*-Value
			Total Control (*n* = 22)	No HF (*n* = 10)	Compensated HF (*n* = 12)	
LVEDD, mm (mean ± SD)	54.61 ± 7.78	57.11 ± 7.25	51.54 ± 7.44	48.20 ± 4.56	54.33 ± 8.38	0.011
LVESD, mm (mean ± SD)	42.20 ± 9.47	44.91 ± 9.88	38.78 ± 7.99	34.75 ± 3.45	41.72 ± 9.04	0.034
LVEF, % (mean ± SD)	37.65 ± 17.45	29.81 ± 14.37	47.27 ± 16.26	59.50 ± 2.83	37.08 ± 15.79	<0.001
LAD, mm (mean ± SD)	45.73 ± 9.61	48.52 ± 10.01	42.32 ± 8.07	39.70 ± 8.47	44.50 ± 7.36	0.023
LAA, cm^2^ (mean ± SD)	26 ± 7.81	28.80 ± 8.45	22.57 ± 5.34	20.94 ± 3.26	23.92 ± 6.42	0.004
RVD, mm (mean ± SD)	33.82 ± 5.99	35.15 ± 6.21	32.18 ± 5.40	31.30 ± 4.47	32.92 ± 6.17	0.085
sPAP, mmHg (mean ± SD)	36.08 ± 23.58	44.11 ± 22.57	26.23 ± 21.35	15.40 ± 7.66	27 ± 20.42	0.007
MAPSE, mm (mean ± SD)	12.35 ± 3.10	11.37 ± 2.84	13.55 ± 3.05	15.00 ± 1.15	12.33 ± 3.62	0.013
Moderate/severe MR, *n* (%)	28 (57.1%)	22 (81.4%)	6 (27.2%)	0 (0%)	6 (50%)	0.002
Moderate/severe TR, *n* (%)	28 (57.1%)	19 (70.4%)	9 (40.9%)	1 (10%)	8 (66.7%)	0.048
E/e’ >15, *n* (%)	15 (32.6%)	12 (50%)	3 (13.6%)	0 (0%)	3 (25%)	0.027

Abbreviations: AHF—acute heart failure; HF—heart failure; *n*—number of patients; y—years; SD—standard deviation; LVEDD—left ventricle end-diastolic diameter; LVESD—left ventricle end-systolic diameter; LVEF—left ventricle ejection fraction; LAD—left atrium diameter; LAA—left atrium area; RVD—right ventricle diameter; sPAP—systolic pulmonary artery pressure; MAPSE—mitral annular plane systolic excursion; MR—mitral regurgitation; TR—tricuspid regurgitation.

**Table 4:** AHF classifications.

Etiology, *n* (%)	
Ischemic disease	15 (55.6%)
Alcoholic DCM	4 (14.8%)
Valvular	5 (18.5%)
Hypertensive heart disease	3 (11.1%)
Ejection fraction, *n* (%)	
HFrEF	24 (88.9%)
HFpEF	3 (11.1%)
HFmrEF	0 (0%)
NYHA Class, *n* (%)	
NYHA I	0 (0%)
NYHA II	6 (22.2%)
NYHA III	16 (59.3%)
NYHA IV	5 (18.5%)
Clinical presentations of AHF, *n* (%)	
Acute decompensated HF	7 (25.9%)
Acute pulmonary oedema	17 (63%)
Cardiogenic shock	3 (11.1%)

Abbreviations: *n*—number of patients; AHF—acute heart failure; DCM—dilated cardiomyopathy; HFrEF—heart failure with reduced ejection fraction; HFpEF—heart failure with preserved ejection fraction; HFmrEF—heart failure with mildly reduced ejection fraction; NYHA—New York Heart Association; HF—heart failure.

**Table 5:** Plasma levels of cardiac biomarkers in relation to acute etiology for AHF.

Biomarker	Acute Coronary Syndrome *n* = 8 (29.6%)	Hypertension Emergency *n* = 6 (22.2%)	Arrhythmia *n* = 4 (14.8%)	Infection *n* = 6 (22.2%)	*p*-Value
MyBP-C, ng/L (median, IQR)	68.51 (38.90–448.94)	4.80 (0.01–93.06)	81.05 (36.47–153.73)	52.33 (12.14–173.21)	0.540
NT-proBNP, pg/mL (median, IQR)	11,969 (3792.50–16,896)	16,753.50 (6346–24,999.75)	8813 (5875.75–15,300.75)	18,448.50 (6972.50–23,847.25)	0.456
hs-TnI, ng/L (median, IQR)	13,918.50 (4000.25–27,812)	91.15 (48.87–446.50)	76.75 (23.27–1222.75)	173 (13.34–661.75)	0.012

Abbreviations: *n*—number of patients; IQR—interquartile range; MyBP-C—myosin binding protein C; NT-proBNP—N-terminal pro b-type natriuretic peptide; hs-TnI—high-sensitivity troponin I.

**Table 6:** Plasma levels of cardiac biomarkers in AHF in different types of EF.

Biomarker	Reduced EF *n* = 24 (88.9%)	Preserved EF *n* = 3 (11.1%)	*p*-Value
MyBP-C, ng/L (median, IQR)	47.46 (6.43–117.23)	64.77 (11.26–321.23)	0.537
NT-proBNP, pg/mL (median, IQR)	14,357 (7345.25–20,562)	17,359 (7031–25,600)	0.643
hs-TnI, ng/L (median, IQR)	276 (53.12–4230.75)	370 (6.87–1597)	0.700

Abbreviations: EF—ejection fraction; *n*—number of patients; MyBP-C—myosin binding protein C; NT-proBNP—N-terminal pro b-type natriuretic peptide; hs-TnI—high-sensitivity troponin I; IQR—interquartile range.

**Table 7:** Plasma levels of cardiac biomarkers in different clinical presentations of AHF.

Biomarker	Acute Decompensated HF *n* = 7 (25.9%)	Acute Pulmonary Edema *n* = 17 (63%)	Cardiogenic Shock *n* = 3 (11.1%)	*p*-Value
MyBP-C, ng/L (median, IQR)	56.84 (5.23–172.54)	35.76 (7.48–69.02)	513.30 (318.58–528)	<0.001
NT-proBNP, pg/mL (median, IQR)	11,268 (8500–29,429)	15,783 (6914–20,011)	17,174 (14,090.50–20,218.50)	<0.001
hs-TnI, ng/L (median, IQR)	349 (19.90–11,841)	143 (47.90–1117)	15,996 (8099.50–22,911.50)	<0.001

Abbreviations: *n*—number of patients; AHF—acute heart failure; MyBP-C—myosin binding protein C; NT-proBNP—N-terminal pro b-type natriuretic peptide; hs-TnI—high-sensitivity troponin I; HF—heart failure; IQR—interquartile range.

**Table 8:** Plasma levels of cardiac biomarkers in different NYHA classes.

Biomarker	Without HF *n* = 10 (20.4%)	Class II NYHA *n* = 6 (22.2%)	Class III NYHA *n* = 16 (59.3%)	Class IV NYHA *n* = 5 (18.5%)	*p*-Value
MyBP-C, ng/L (median, IQR)	0.01 (0.01–0.01)	0.01 (0.01–50.26)	27.04 (0.01–68.94)	97.33 (8.25–431.96)	0.011
NT-proBNP, pg/mL (median, IQR)	53.50 (39.73–73.55)	329 (127.50–9854)	11,268 (3682.50–19,418.50)	11,007 (8078.50–27,800)	<0.001
hs-TnI, ng/L (median, IQR)	2.29 (1.33–2.74)	53 (3.82–1123.50)	107 (17.70–2711)	349 (10.03–15,098.50)	0.006

Abbreviations: NYHA—New York Heart Association; HF—heart failure; MyBP-C—myosin binding protein C; NT-proBNP—N-terminal pro b-type natriuretic peptide; hs-TnI—high-sensitivity troponin I; IQR—interquartile range.

**Table 9:** Plasma levels of cardiac biomarkers in patients with AHF and AMI.

Biomarker	AMI *n* = 9 (33.3%)	Non-AMI *n* = 18 (66.6%)	*p*-Value
MyBP-C, ng/L (median, IQR)	80.18 (44.23–384.58)	31.40 (4.92–79.29)	0.046
NT-proBNP, pg/mL (median, IQR)	12,931 (5169–20,311)	15,783.50 (7122.50–21,668)	0.668
hs-TnI, ng/L (median, IQR)	11,841 (1123.50–25,797)	87.65 (32.35–364.75)	<0.001

Abbreviations: *n*–number of patients; AHF—acute heart failure; AMI—acute myocardial infarction; MyBP-C—myosin binding protein C; NT-proBNP—N-terminal pro b-type natriuretic peptide; hs-TnI—high-sensitivity troponin I; IQR—interquartile range.

**Table 10:** Correlations between MyBP-C serum levels and relevant parameters.

Parameter	My-BPC		NT-proBNP		hs-TnI	
	*r*	*p*-Value	*r*	*p*-Value	*r*	*p*-Value
Age	0.209	0.149	0.456	0.001	0.348	0.014
Gender	0.093	0.525	0.217	0.134	0.082	0.574
Alcohol abuse	0.176	0.226	0.008	0.956	0.105	0.474
Smoking	0.012	0.933	−0.122	0.404	−0.056	0.703
Obesity	−0.028	0.851	−0.218	0.133	−0.070	0.634
Dyspnea	0.707	<0.001	0.837	<0.001	0.644	<0.001
Pulmonary rales	0.597	<0.001	0.602	<0.001	0.500	<0.001
SBP	−0.085	0.563	0.291	0.042	0.132	0.364
DBP	−0.226	0.119	0.170	0.242	0.029	0.843
HR	0.136	0.350	0.258	0.074	0.279	0.052
Oxygen saturation	−0.487	<0.001	−0.618	<0.001	−0.623	<0.001
Lung congestion	0.681	<0.001	0.612	<0.001	0.539	<0.001
AMI	0.535	<0.001	0.332	0.020	0.607	<0.001
PE	−0.216	0.135	−0.296	0.039	−0.381	0.007
Infection	0.214	0.140	0.239	0.099	0.114	0.434
Sepsis	0.228	0.115	0.311	0.030	0.258	0.073
Atrial fibrillation	0.456	0.001	0.435	0.002	0.452	0.001
Anemia	0.256	0.076	0.262	0.069	0.146	0.318
CKD	0.148	0.315	0.355	0.013	0.074	0.617
COPD	0.196	0.177	0.303	0.034	0.412	0.003
Diabetes mellitus	0.189	0.193	0.164	0.259	0.167	0.250
Cardiogenic shock	0.413	0.003	0.247	0.087	0.307	0.032
Inotropic support	0.367	0.009	0.184	0.204	0.158	0.278
Loop diuretic dose	0.574	<0.001	0.731	<0.001	0.599	<0.001
Invasive ventilation	0.440	0.002	0.295	0.040	0.353	0.013
Inability of selfcare	−0.274	0.057	−0.446	0.001	−0.190	0.190
In-hospital mortality	0.483	<0.001	0.300	0.036	0.300	0.036
Death within 30 days	0.412	0.003	0.329	0.021	0.074	0.614
Length of hospital stay	0.333	0.019	0.236	0.102	0.127	0.383
Rehospitalization within 30 days	0.606	<0.001	0.361	0.011	0.481	<0.001

Abbreviations: MyBP-C—myosin binding protein C; HR—heart rate; SBP—systolic blood pressure; DBP—diastolic blood pressure; AMI—acute myocardial infarction; PE—pulmonary embolism; CKD—chronic kidney disease; COPD—chronic obstructive pulmonary disease.

**Table 11:** Correlations between MyBP-C serum levels and laboratory and echocardiographic parameters.

Parameter	My-BPC		NT-proBNP		hs-TnI	
	*r*	*p*-Value	*r*	*p*-Value	*r*	*p*-Value
C-reactive protein	0.343	0.016	0.280	0.052	0.305	0.033
Hemoglobin	−0.266	0.065	−0.283	0.049	−0.072	0.621
Leucocytes	0.265	0.065	0.480	<0.001	0.321	0.025
Serum iron	−0.269	0.065	−0.335	0.020	−0.205	0.162
Ferritin	0.105	0.515	−0.032	0.843	−0.079	0.623
Serum creatinine	0.453	0.001	0.495	<0.001	0.392	0.005
eGFR	−0.483	<0.001	−0.595	<0.001	−0.430	0.002
UNa+	−0.426	0.002	−0.447	0.001	−0.337	0.018
ACR	0.385	0.006	0.733	<0.001	0.468	0.001
Sodium	−0.300	0.036	−0.233	0.108	−0.213	0.142
Lactate	0.132	0.465	0.014	0.939	0.438	0.011
Serum bicarbonate	−0.489	0.001	−0.202	0.211	−0.198	0.221
Albumin	−0.332	0.020	−0.283	0.049	−0.205	0.158
Uric acid	0.449	0.002	0.453	0.002	0.158	0.293
AST	0.389	0.006	0.465	0.001	0.621	<0.001
ALT	0.104	0.481	0.206	0.160	0.207	0.158
Total bilirubin	0.239	0.098	0.213	0.143	0.183	0.208
TSH	0.028	0.850	0.068	0.647	0.033	0.824
Total cholesterol	−0.181	0.218	−0.116	0.432	−0.071	0.632
Glycemia	0.047	0.753	0.174	0.242	0.255	0.083
pH	−0.098	0.582	0.000	0.999	−0.188	0.286
LVEDD	0.289	0.044	0.355	0.012	0.379	0.007
LVEF	−0.399	0.004	−0.554	<0.001	−0.540	<0.001
LAA	0.367	0.010	0.435	0.002	0.149	0.306
RVD	0.177	0.223	0.205	0.157	0.251	0.081
sPAP	0.449	0.001	0.582	<0.001	0.413	0.003
MAPSE	−0.348	0.014	−0.546	<0.001	−0.386	0.006
TAPSE	−0.196	0.176	−0.277	0.054	−0.258	0.073
E/e′ > 15	0.327	0.022	0.449	0.001	0.357	0.012
LV systolic dysfunction	0.334	0.019	0.539	<0.001	0.551	<0.001
moderate/severe MR	0.403	0.004	0.563	<0.001	0.525	<0.001
moderate/severe TR	0.277	0.054	0.429	0.002	0.315	0.028
IVC	0.355	0.012	0.425	0.002	0.386	0.006

Abbreviations: MyBP-C—myosin binding protein C; ACR—albumin creatinin ratio; UNa+—urinary sodium; eGFR—estimated glomerular filtration rate; AST—aspartate transaminase; ALT—alanine transaminase; TSH—thyroid stimulating hormone; CK-MB—creatine kinase myocardial band; NT-proBNP—N-terminal pro b-type natriuretic peptide; hs-TnI—high-sensitivity troponin I; pH—potential of hydrogen; LVEDD—left ventricle end-diastolic diameter; LVEF—left ventricle ejection fraction; LAA—left atrium area; RVD—right ventricle diameter; sPAP—systolic pulmonary artery pressure; MAPSE—mitral annular plane systolic excursion; TAPSE—tricuspid annular plane systolic excursion; LV—left ventricle; IVC—inferior vena cava.

**Table 12:** Correlations between MyBP-C serum levels and standard cardiac biomarkers.

Parameter	My-BPC	
	*r*	*p*-Value
NT-proBNP	0.727	<0.001
hs-TnI	0.604	<0.001

Abbreviations: MyBP-C—myosin binding protein C; NT-proBNP—N-terminal pro b-type natriuretic peptide; hs-TnI—high-sensitivity troponin I.

**Table 13:** Detailed analysis of AUC: diagnostic performance of MyBP-C compared to NT-proBNP and hs-TnI for AHF.

Biomarker	AUC	Std. Error	Asymptotic 95% Confidence Interval		*p*-Value
			Lower Bound	Upper Bound	
MyBP-C (ng/L)	0.924	0.040	0.841	1.00	<0.001
NT-proBNP (pg/mL)	0.998	0.003	0.000	1.00	<0.001
hs-TnI (ng/L)	0.912	0.047	0.813	1.00	<0.001

Abbreviations: AHF—acute heart failure; MyBP-C—myosin binding protein C; NT-proBNP—N-terminal pro b-type natriuretic peptide; hs-TnI—high-sensitivity troponin I; Std. Error—standard error; AUC—area under the curve.

**Table 14:** Comparison of characteristics between patients dying during the 30-days follow-up with those surviving.

Characteristic	Mortality (*n* = 9)	No Mortality (*n* = 18)	*p*-Value
Age, y (mean ± SD)	65.67 ± 8.68	74.39 ± 9.29	0.027
Male Gender (%)	4 (44.4%)	11 (61.1%)	0.411
Length of hospital stay, days (mean ± SD)	12.75 ± 5.90	10 ± 4.82	0.873
SBP, mmHg (mean ± SD)	170 ± 54.94	168.17 ± 36.48	0.124
HR, beats/minute (mean ± SD)	114.25 ± 17.67	95.33 ± 23.32	0.970
NYHA functional class IV (%)	3 (33.3%)	2 (11.1%)	0.161
Smoking (%)	3 (33.3%)	7 (38.9%)	0.778
Obesity (%)	4 (44.4%)	7 (38.9%)	0.782
Diabetes mellitus (%)	5 (55.6%)	8 (44.4%)	0.586
Arterial hypertension (%)	7 (77.8%)	16 (88.9%)	0.444
Atrial fibrillation (%)	8 (88.9%)	9 (50%)	0.049
Anemia (%)	4 (44.4%)	8 (44.4%)	1
Myocardial infarction (%)	4 (44.4%)	5 (27.8%)	0.756
COPD (%)	1 (11.1%)	5 (27.8%)	0.326
CKD (%)	9 (100%)	15 (83.3%)	0.194
NT-proBNP, pg/mL (median, IQR)	17,174 (10,066.50–24431.50)	12,099.50 (6348–19,460)	0.173
MyBP-C, ng/L (median, IQR)	152.44 (81.05–417.26)	19.73 (4.92–55.37)	<0.001
hs-TnI, ng/L (median, IQR)	203 (34.50–8796.50)	356 (50.45–4000.25)	0.797
CRP, mg/dL (mean± SD)	3.88 ± 3.19	2.47 ± 2.60	0.229
Creatinine, mg/dL (mean ± SD)	1.94 ± 1.15	1.24 ± 0.37	0.155
ACR, mg/g (median, IQR)	127.95 (59.71–1725.08)	83.50 (23.01–176.95)	0.280
UNa^+^, mEq/L (mean ± SD)	57.25 ± 43.53	69.33 ± 30.09	0.129
eGFR, mL/min/1.73 m^2^ (mean ± SD)	47.75 ± 32.26	61.08 ± 22.94	0.093
Uric acid, mg/dL (mean ± SD)	9.62 ± 0.20	7.90 ± 2.41	0.029
Serum iron, mcg/dL (mean ± SD)	46.50 ± 30.88	35 ± 13.54	0.728
Ferritin, ng/mL (mean ± SD)	233.25 ± 230.22	318.67 ± 567.41	0.644
Albumin, g/dL (mean ± SD)	52.31 ± 6.63	58.42 ± 5.79	0.021
Lactate, mmol/L (mean ± SD)	5.02 ± 4.64	1.97 ± 0.68	0.263
Serum bicarbonate, mEq/L (mean ± SD)	18.97 ± 3.54	21.30 ± 2.24	0.042
LVEF, % (mean ± SD)	30 ± 23.45	35 ± 12.79	0.927
sPAP, mmHg (mean ± SD)	34 ± 12.72	47.17 ± 20.76	0.186
LAA, mm^2^ (mean ± SD)	32.66 ± 8.77	26.48 ± 5.70	0.032
MAPSE, mm (mean ± SD)	9 ± 3.36	12.33 ± 2.74	0.239
E/e′ > 15 (%)	5 (55.6%)	7 (46.7%)	0.411

Abbreviations: *n*—number of patients; y—years; SD—standard deviation; IQR—interquatile range; SBP—systolic blood pressure; HR—heart rate; NYHA—New York Heart Association; COPD—chronic obstructive pulmonary disease; CKD—chronic kidney disease; MyBP-C—myosin binding protein C; NT-proBNP—N-terminal pro b-type natriuretic peptide; hs-TnI—high-sensitivity troponin I; CRP—C reactive protein; ACR—albumin creatinin ratio; UNa+—urinary sodium; eGFR—estimated glomerular filtration rate; LVEF—left ventricle ejection fraction; sPAP—systolic pulmonary artery pressure; LAA—left atrium area; MAPSE—mitral annular plane systolic excursion.

**Table 15:** Detailed analysis of AUC: diagnostic performance of MyBP-C compared to NT-proBNP and hs-TnI for mortality within 30 days.

Biomarker	AUC	Std. Error	Asymptotic 95% Confidence Interval		*p*-Value
			Lower Bound	Upper Bound	
MyBP-C (ng/L)	0.972	0.021	0.000	1	<0.001
NT-proBNP (pg/mL)	0.849	0.054	0.742	0.955	0.001
hs-TnI (ng/L)	0.714	0.078	0.560	0.868	0.047

Abbreviations: HF—heart failure; MyBP-C—myosin binding protein C; NT-proBNP—N-terminal pro b-type natriuretic peptide; hs-TnI—high-sensitivity troponin I; Std. Error—standard error; AUC—area under the curve.

**Table 16:** Detailed analysis of AUC: diagnostic performance of MyBP-C compared to NT-proBNP and hs-TnI for HF rehospitalization within 30 days.

Biomarker	AUC	Std. Error	Asymptotic 95% Confidence Interval		*p*-Value
			Lower Bound	Upper Bound	
MyBP-C (ng/L)	0.897	0.046	0.806	0.987	<0.001
NT-proBNP (pg/mL)	0.750	0.070	0.614	0.886	0.012
hs-TnI (ng/L)	0.833	0.060	0.715	0.950	0.001

Abbreviations: HF—heart failure; MyBP-C—myosin binding protein C; NT-proBNP—N-terminal pro b-type natriuretic peptide; hs-TnI—high-sensitivity troponin I; Std. Error—standard error; AUC—area under the curve.

**Table 17:** Overall survival assessment between subgroups according to the high-risk cut-off (64.69 ng/L).

	Chi-Square	df	*p*-Value
Log Rank (Mantel–Cox)	35.191	1	<0.001
Test of equality of survival distributions for the different levels of MyBP-C			

**Table 18:** Adjusted multivariate analysis for death within 30 days and for the composite of death/recurrent HF within 30 days.

Predictor	Odd Ratio	95% Confidence Interval	*p*-Value
Death within 30 days			
MyBP-C	1.08	1.0–1.16	0.039
NT-proBNP	1.0	1.0–1.0	0.49
hs-TnI	1.0	0.99–1.0	0.09
Composite of Death/Recurrent HF within 30 days			
MyBP-C	1.12	1.02–1.22	0.014
NT-proBNP	1.0	1.0–1.0	0.33
hs-TnI	1.0	1.0–1.0	0.48

Abbreviations: HF—heart failure; MyBP-C—myosin binding protein C; NT-proBNP—N-terminal pro b-type natriuretic peptide; hs-TnI—high-sensitivity troponin I.

## Data Availability

The data presented in this study are available within the article.
